# The Use of Presurgical Lip–Alveolus–Nose Approximation in Early Infant Orthopedics: A Clinical Case Series

**DOI:** 10.3390/medicina62081605

**Published:** 2026-08-21

**Authors:** Neda Najafimakhsoos, Rene Myers, Shelby Svientek, Cassendra Smola, Nathaniel H. Robin, Kathlyn Kruger Powell, Chung How Kau

**Affiliations:** 1 Department of Biomedical and Neuromotor Sciences, University of Bologna, 40125 Bologna, Italy; neda.najafimakhsoos@studio.unibo.it; 2 Division of Plastic Surgery, Department of Surgery, Heersink School of Medicine, University of Alabama at Birmingham, Birmingham, AL 35294, USA; rmyers@uabmc.edu (R.M.); surgerysrsvientek@uabmc.edu (S.S.); 3 Division of Hospital Medicine, Department of Pediatrics, Heersink School of Medicine, University of Alabama at Birmingham, Birmingham, AL 35294, USA; csmola@uabmc.edu; 4 Department of Genetics, Heersink School of Medicine, University of Alabama at Birmingham, Birmingham, AL 35294, USA; nrobin@uabmc.edu; 5 Department of Oral and Maxillofacial Surgery, School of Dentistry, University of Alabama at Birmingham, Birmingham, AL 35294, USA; krugelk@uab.edu; 6 Department of Orthodontics, School of Dentistry, University of Alabama at Birmingham, Birmingham, AL 35294, USA; 7 Department of Surgery, Heersink School of Medicine, University of Alabama at Birmingham, Birmingham, AL 35294, USA

**Keywords:** presurgical infant orthopedics, PSIO, presurgical lip–alveolus–nose approximation, PLANA, cleft lip and palate, unilateral cleft, bilateral cleft, nasoalveolar molding, NAM, nasal molding, nasolabial morphology, nasal symmetry, presurgical cleft management

## Abstract

*Background and Objectives:* Presurgical infant orthopedics aims to reduce cleft severity and facilitate primary surgical repair during the early neonatal period, when increased tissue plasticity enhances molding. Nasoalveolar molding is widely used but requires intraoral appliances, laboratory support, specialized training, and frequent follow-up visits, which may limit accessibility and adherence. This case series reports the clinical application of presurgical lip–alveolus–nose approximation (PLANA), an extraoral technique for early presurgical cleft management, and describes the clinical changes observed during treatment in four infants. *Materials and Methods:* A prospective case series conducted in neonates with non-syndromic unilateral or bilateral cleft lip and/or palate referred within the first 10 days of life to the Alabama Cleft Center. Treatment consisted of a prefabricated silicone nasal aligner combined with hydrocolloid lip adhesive taping. No intraoral plates or dental impressions were used. Devices were worn 20 to 22 h daily. Follow up occurred every 2 to 4 weeks, with remote monitoring used when appropriate. Qualitative clinical observations focused on apparent changes in nasal symmetry, columellar length, septal alignment, nasal tip projection, premaxillary position, nostril dimensions, alar base width, cleft width, and lip approximation, as well as feeding tolerance, skin tolerance, and treatment adherence. These observations were assessed qualitatively through serial clinical examinations and clinical photographs; no standardized quantitative morphometric measurements were performed. *Results:* Four infants with complete unilateral or bilateral cleft lip and palate received PLANA treatment. Serial clinical examinations and photographs documented qualitative changes in nasolabial morphology over the course of treatment in all four patients. Qualitative clinical assessment of serial photographs and examinations suggested greater apparent nasal symmetry, apparent columellar elongation, more central-appearing nasal alignment, increased apparent nasal tip projection, apparent cleft-width reduction, and progressive lip approximation. In unilateral clefts, changes were observed in both the cleft and non-cleft nasal sides, while bilateral clefts demonstrated changes in nasal morphology bilaterally. No device-related complications or clinically significant skin intolerance were documented during the reported treatment periods. Parent-reported device wear was approximately 20–22 h per day. *Conclusions:* In this four-patient prospective descriptive case series, PLANA was applied clinically, with no clinically significant treatment-related complications documented during the reported treatment periods. Serial clinical observations suggested changes in nasolabial morphology during treatment. Given the small sample size, absence of a contemporaneous control group, and qualitative nature of the assessments, these findings should be interpreted as preliminary and hypothesis-generating rather than as evidence of treatment efficacy or comparative effectiveness. Larger prospective, multicenter comparative studies incorporating standardized quantitative measurements, objective adherence assessment, and long-term surgical and aesthetic outcomes are needed to evaluate the effectiveness, reproducibility, and clinical applicability of the PLANA approach.

## 1. Introduction

The management of cleft patients requires long-term, multidisciplinary care. At a minimum, the core team should include professionals from speech-language pathology, surgery, and orthodontics, who participate in team meetings according to the specific needs of each patient [1].

Presurgical infant orthopedics (PSIO) dates back to 1689 with Hoffmann’s early techniques [2], while its modern form began in 1950 with McNeil [3]. PSIO is applied before cleft lip and/or palate (CLP) surgery to approximate the tissues around the cleft [4], reduce its severity, enhance maxillary arch form, and reposition the alar base [3,5]. These objectives contribute to preventing crossbites, improving surgical outcomes, facilitating the surgical procedure, and minimizing postoperative scarring [4]. It also supports better feeding to prevent nutritional deficiencies, enhances speech development, improves facial aesthetics and maxillofacial growth, and helps prevent complications caused by muscle dysfunction or nasal breathing issues [6,7]. These complications may include reduced upper facial height, retruded maxilla, hyperdivergent growth patterns, retroclined incisors, obtuse nasolabial and interincisal angles, posterior crossbite, and class III malocclusion [8]. Additionally, PSIO helps address parents’ concerns regarding their child’s function, health, and appearance. In the first six weeks after birth, especially during the first two weeks, high levels of maternal estrogen in the fetal circulation lead to elevated hyaluronic acid, making nasal cartilage, alveolar bone, and soft tissues highly moldable [9,10]. Hyaluronic acid increases tissue plasticity by altering the elasticity of cartilage, ligaments, and connective tissue through breakdown of the intercellular matrix [9,10], facilitating easier molding of both bone and soft tissue. This creates a unique and ideal window for nonsurgical molding of nasal cartilage and alveolar segments [9,10].

Several types of PSIO therapies are used, including lip taping, passive and active oral plates, nasoalveolar molding (NAM), and lip adhesion surgery [11]. Lip adhesion is a surgical procedure that converts a complete cleft into an incomplete one by narrowing the cleft gap often as a staged procedure. However, it carries risks such as wound dehiscence and potential reoperation [12]. Lip taping, on the other hand, is a non-surgical technique that uses adhesive tape to approximate the cleft segments, though it may cause skin irritation or lose effectiveness due to tape stretching and relies on parental compliance [12]. Active devices, such as the Latham appliance, which is surgically inserted, utilize mechanical forces, including screws, elastics, or wires, to bring the palatal shelves together and reshape the cleft anatomy. However, they are associated with post-placement discomfort and potential damage to tooth buds [12]. One concern is the potential inhibition of maxillary growth, possibly affecting future dentition, though no causal relationship was confirmed [13]. The Latham appliance remains controversial: one study [14] reported improved nasolabial esthetics when used with gingivoperiosteoplasty, but noted significant midface retrusion, while another study [15] found no adverse effect on midfacial growth.

Passive appliances, such as feeding plates and the Hotz appliance, are commonly used [12]. They do not apply direct force but instead act by preventing tongue interference in the cleft area, allowing natural movement of the palatal shelves to reduce the cleft gap while maintaining oral function [16]. However, their effectiveness remains questionable. A study by Prahl et al. on patients with unilateral cleft lip and palate found that passive PSIO neither approximated the maxillary segments nor prevented alveolar collapse, showing no significant benefit over no treatment [6]. These results are supported by other studies, which also found no meaningful difference between passive PSIO and no intervention [6,16]. Additionally, passive appliances fail to improve alveolar alignment or facial appearance. Different studies have reported that PSIO reduces anterior and canine crossbites and improves maxillary arch dimensions [7,17,18]. However, these effects are temporary, and by the age of five, patients treated with PSIO show the same number and severity of teeth in crossbite as those who did not receive the treatment [5,18].

Addressing nasal deformities remains a major challenge in cleft care [15], which led to the development of nasoalveolar molding (NAM) to enhance facial esthetics in the nasoalveolar region [16]. NAM is currently the most widely used PSIO technique in North America [11]. However, it presents several limitations. It requires dental impressions and includes an intraoral component, making adaptation difficult for some neonates. This is especially so when the NAM is not well constructed and it can cause soft tissue injuries, feeding difficulties, and general discomfort. NAM can be costly and require a dental laboratory, specialized clinician training, and weekly follow-up visits. The follow-up visits may be difficult for families living far from cleft centers, and the need to make residential arrangements can be challenging [11]. Parents must also be trained to handle and tape the device properly. These factors contribute to reduced treatment adherence, which is essential for NAM’s effectiveness [11]. As discussed, early nasal cartilage molding is critical due to high hyaluronic acid levels in neonates, but use of NAM may delay the use of this critical window. The nasal stent can only be inserted once the alveolar segments are within 4–5 mm, delaying nasal correction [11]. Even when applied correctly, NAM can cause hard and soft tissue complications [19]. Hard tissue problems include alveolar segment misalignment, forming a T-shaped maxillary arch or a “locked-out” segment—where the greater segment moves backward faster than the lesser segment expands forward [19]. This may trap the lesser segment behind. Premature eruption of maxillary incisors or neonatal teeth, caused by plate pressure, may require extraction if teeth are supernumerary, mobile, or ectopic [19]. Soft tissue complications may include gum bleeding, irritation, ulceration, inflammation of the intranasal lining at the nasal tip due to excessive pressure from the nasal stent, petechiae caused by high pressure or over-expanded nostrils, columellar bleeding or tearing, oral candidiasis, and tissue breakdown at the labial frenulum insertion site or in the posterior fauces during retraction of the molding plate [19]. Treating unilateral clefts is particularly challenging. Since the nasal stent is applied only to the cleft-side nostril, it can cause over-elongation and alter the nostril height ratio between the cleft and non-cleft side.

A novel technique using a prefabricated nasal stent, lip silicone bar, and external lip taping was recently introduced. This technique, known as presurgical lip–alveolus–nose approximation (PLANA), is designed to address many of NAM’s limitations. Unlike NAM, PLANA does not use an intraoral palatal plate, potentially making it easier for infants to tolerate and eliminating the risk of soft tissue injuries [11]. It also does not require dental impressions or laboratory fabrication. Follow-up appointments are needed only every 2–4 weeks, which is particularly beneficial for families living far from cleft centers, and it also allows for remote monitoring, significantly reducing the treatment burden [11]. PLANA enables earlier initiation of nasal correction, helping to minimize the severity of nasal deformity. It utilizes a bilateral nasal aligner that applies uniform molding forces to both nostrils, encouraging more symmetrical outcomes between cleft and non-cleft sides while minimizing the risk of overexpansion. Once the desired nasal cartilage molding is achieved, the nasal aligner functions passively [1].

Given the limited published clinical experience with PLANA, including only one previous clinical study [11], we present a descriptive case series of four infants treated with this technique. The purpose of this report is to describe the clinical application of PLANA, the treatment procedures, and the qualitative clinical changes observed during presurgical management. We also describe practical aspects of implementing the protocol in these cases. This report provides preliminary descriptive clinical experience and identifies observations that may inform the design of future prospective studies evaluating PLANA using standardized quantitative outcome measures, appropriate comparison groups, and longer-term follow-up.

## 2. Materials and Methods

### 2.1. Study Design and Patient Population

This prospective, descriptive case series included infants with unilateral or bilateral cleft lip and/or cleft palate who were referred to the University of Alabama at Birmingham (UAB) Alabama Cleft Craniofacial Center between 7 and 10 days of age during neonatal period.

Inclusion criteria were neonates with nonsyndromic unilateral or bilateral cleft lip and/or palate, younger than 2 weeks at initiation of therapy, who had not received previous presurgical orthopedic intervention before referral to the Alabama Cleft Craniofacial Center. Exclusion criteria included syndromic cases, previous presurgical interventions, and patients presenting after the optimal age window for PSIO.

All eligible patients received PSIO treatment using the PLANA technique. Because this was a single-arm case series, neither a contemporaneous nor a historical NAM control group was included. Accordingly, any discussion of NAM in this manuscript is limited to contextual comparisons with outcomes reported in the previously published literature and does not represent a direct within-study comparison. The present study evaluated the presurgical orthopedic intervention and does not report outcomes related to subsequent surgical repair of the cleft.

### 2.2. Institutional Review Board Approval

This study was reviewed and approved by the University of Alabama at Birmingham Institutional Review Board (IRB-300011746; expedited review; approval date: 20 November 2024; expiration date: 19 November 2027).

### 2.3. PLANA Presurgical Infant Orthopedic Technique

The PLANA technique consists of three prefabricated extraoral NoseAlign devices, along with LipAlign adhesive tapes, designed to approximate the nasolabial structures. The technique does not require intraoral plates or dental impressions.

The NoseAlign device, crafted from medical-grade silicone, is similar in design to nasal conformers but includes a unique angled horizontal lip band secured with elastic clasps. Infants wear the device for 20 to 22 h per day over a period of 2 to 4 weeks, with sizes adjusted to accommodate facial growth. LipAlign tapes, made from hydrocolloid medical adhesive, are also worn daily for 20 to 22 h and replaced every day. Treatment begins with larger tape sizes and gradually shifts to smaller ones as the cleft gap decreases throughout therapy.

**Figure 1:**
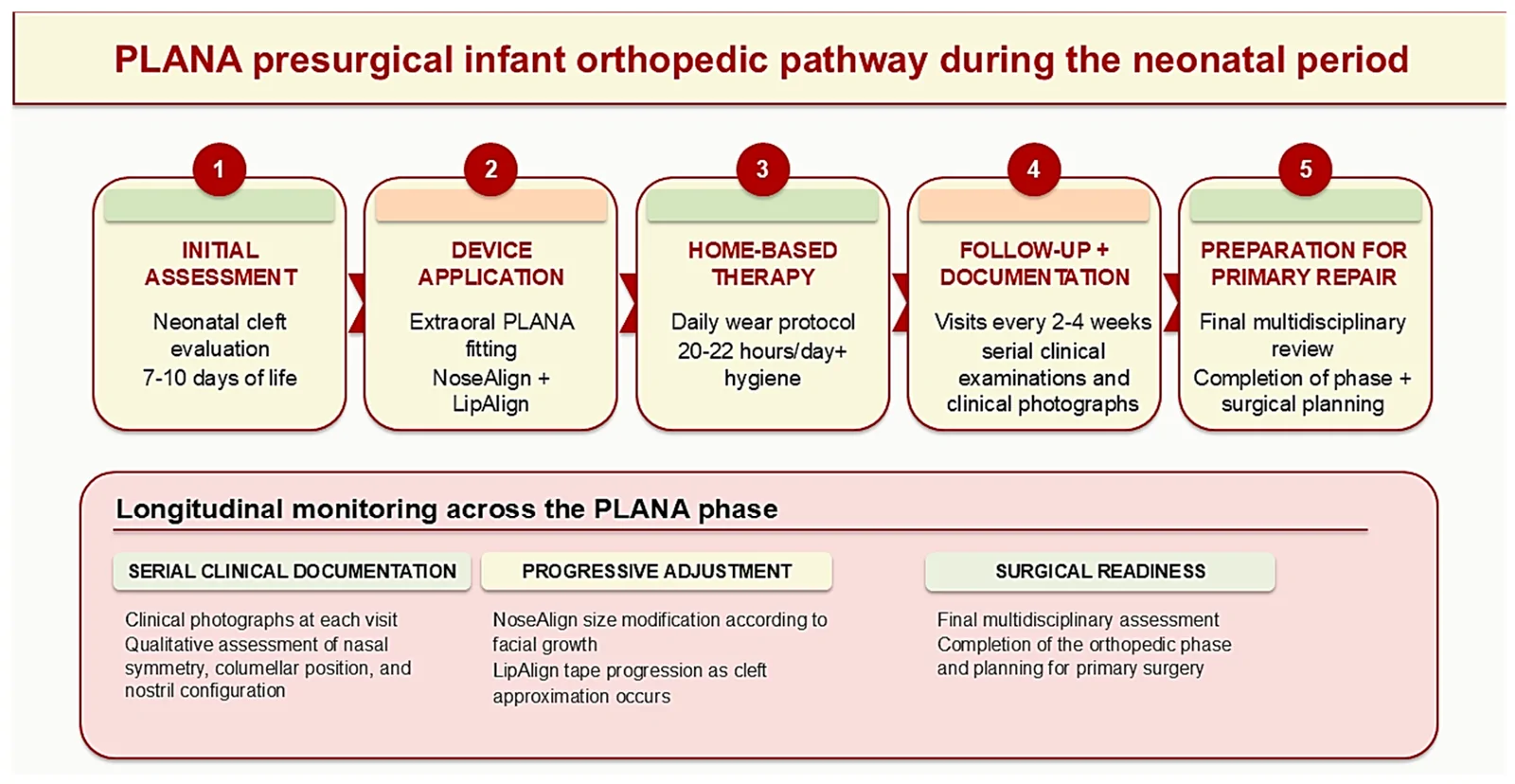
Workflow illustrating the clinical implementation of the PLANA presurgical infant orthopedic protocol. The diagram summarizes the treatment pathway from initial evaluation of neonates with unilateral or bilateral cleft lip and/or palate through PLANA device application, home-care instructions, follow-up monitoring, clinical documentation, and preparation for subsequent cleft surgical care.

The device was applied by trained clinicians, and parents were educated on at-home care, including taping techniques and hygiene. Follow-up visits were scheduled every 2 to 4 weeks, with remote monitoring employed when in-person visits were not feasible. A schematic overview of the PLANA treatment workflow, including initial neonatal assessment, device placement, caregiver education, follow-up monitoring, clinical documentation, and preparation for subsequent cleft care, is presented in Figure 1.

Treatment duration varied depending on the severity of the cleft and the timing of surgical intervention. Clinical observations descriptively documented during treatment included clinically apparent changes in nasal symmetry, columellar position and apparent length, premaxillary alignment, nostril size, nasal septal orientation, cleft width, lip approximation, skin tolerance, feeding tolerance, and infant and parental compliance. The occurrence and type of reversible transient side effects were also recorded.

### 2.4. Clinical Assessment

Clinical assessment of treatment-related anatomical changes was performed by two experienced clinicians (Dr. Kau and Dr. Myers) using serial clinical photographs together with routine clinical examinations. The evaluation focused on qualitative changes in nasolabial morphology, including nasal symmetry, columellar position, nasal contour, nostril shape, lip approximation, and apparent cleft-width reduction. No validated quantitative anthropometric measurements, digital image analysis, or standardized scoring system was used; therefore, the assessments were qualitative and based on clinical judgment.

Formal interobserver and intraobserver reliability assessments were not performed because the present study was designed as a descriptive four-case series based on qualitative clinical observations. Because this study included four patients and was designed as a descriptive qualitative case series without objective quantitative outcome measures, statistical analyses were not performed, as such analyses would not have been methodologically appropriate.

## 3. Results

Four infants met the predefined inclusion criteria and were included in this descriptive case series. The baseline demographic and clinical characteristics of the four infants are presented in Table 1, including cleft type, age at treatment initiation, treatment duration, baseline clinical findings, and documented treatment wear schedule. Table 2 summarizes the qualitatively observed changes in nasal and cleft-related characteristics across the four cases following PLANA therapy. Because this study was designed as a descriptive four-patient case series, the reported outcomes represent qualitative clinical observations documented during routine clinical examinations and serial photographs rather than objectively quantified treatment effects.

### 3.1. Case #1

**Figure 2:**
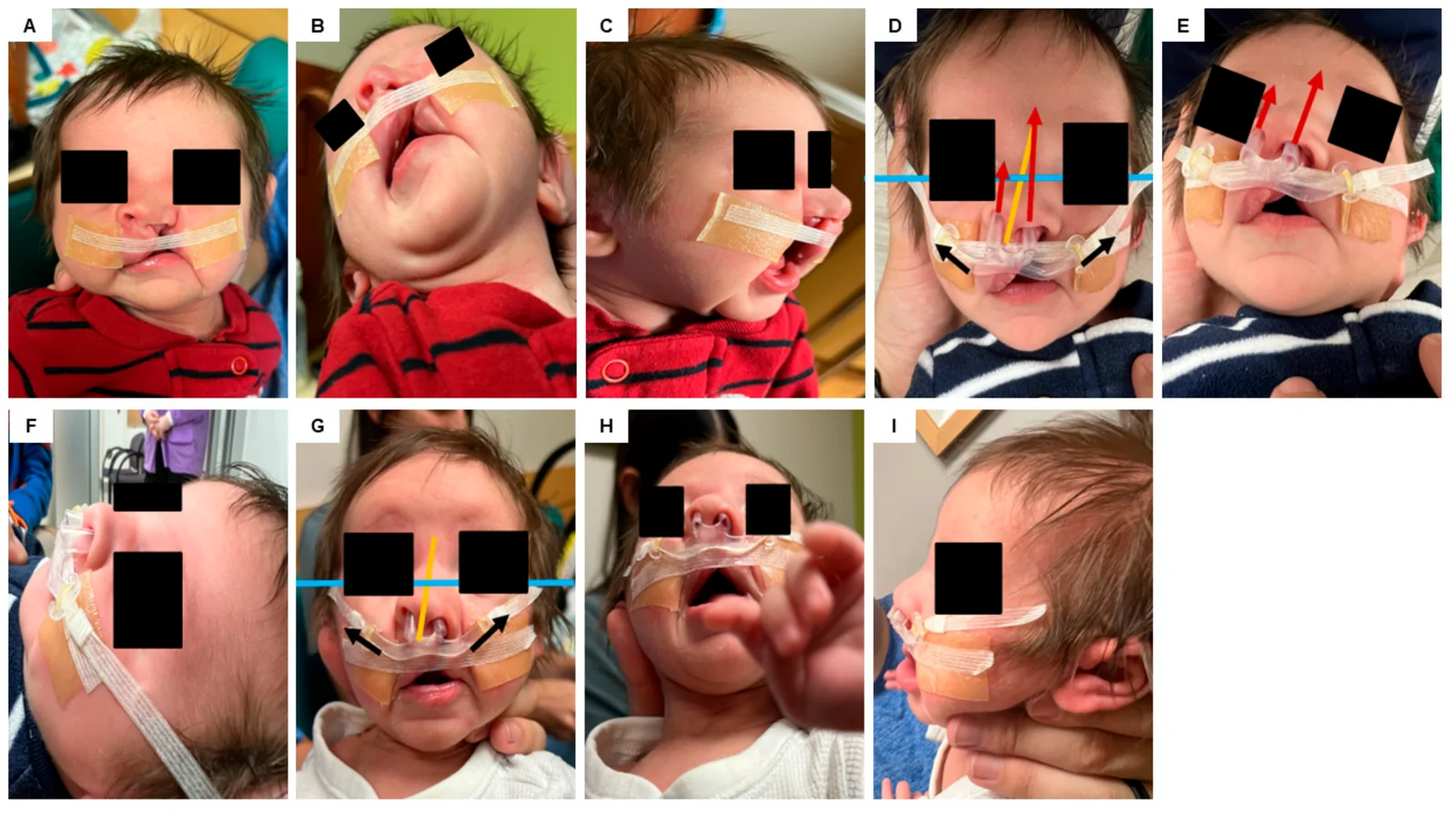
Clinical progression of an infant with unilateral cleft lip and palate treated using the PLANA technique. (**A**–**C**): Initial presentation with LipAlign taping. (**D**,**E**): After 2 weeks of treatment using NoseAlign Size #1 (52.0 × 12.63 × 6.75 mm), supported by 3M Steri-Strips and 3/16-inch, 3.5 oz orthodontic elastics. Note the symmetric elastic pull (black arrows) and the parallel upward-directed forces delivered by the NoseAlign device (red arrows). (**F**–**I**): At 6 weeks of treatment, continued use of NoseAlign Size #1. In (**G**), note the asymmetric elastic pull (black arrows), reflecting an adjustment in the direction of elastic force toward the cleft side.

An infant with left unilateral cleft was referred to the Alabama Cleft Center within the first weeks of life. At initial presentation (Figure 2A–C), the infant exhibited a unilateral cleft, accompanied by distorted nasal cartilage, a deviated nasal septum toward the non-cleft side, and a widened alar base. The cleft side also presented with notable nasal asymmetry, including a flat and laterally displaced alar base, narrowing of the nostril, a shortened columella, and significant soft tissue deficiency. Taping therapy was initiated immediately, with the tape applied to approximate the soft tissue margins and facilitate cleft approximation (Figure 2A–C).

At two weeks of therapy using the NoseAlign device, Size #1 (52.0 × 12.63 × 6.75 mm, NAS 01), combined with daily application of LipAlign hydrocolloid adhesive tapes, early soft tissue changes were observed. The angled lip band with side elastic anchors was positioned to provide gentle, continuous pressure over the nasal and columellar tissues. This setup offered bilateral support, with apparent upright positioning of the columella and a mild reduction in cleft gap width. Early signs of soft tissue molding were also observed. However, nasal dome projection remained limited, and overall nasal symmetry remained limited, with clinically observable asymmetry persisting (Figure 2D–F).

The red parallel arrows indicate the direction of the upward-directed forces applied bilaterally by the nasal stents to the nasal alae. These forces are intended to provide upward support to the nasal alae and columellar region, with the aim of influencing columellar position, alar cartilage configuration, and nasal tip projection and symmetry in the infant with cleft lip and palate (Figure 2D,E).

The yellow line represents the glabella–subnasal line, while the blue line indicates the interpupillary line. In Figure 2D, the black arrows illustrate a symmetrical elastic force originating from the lip alignment device, indicating relatively equal bilateral tension directed toward the midline, as used during the early treatment phase. In contrast, Figure 2G shows an apparent asymmetry in the direction and angle of the black arrows, particularly on the cleft side, where greater force was applied. This configuration represents a later stage of treatment in which the elastic force was adjusted asymmetrically toward the cleft-affected side.

LipAlign tapes were changed daily—initially using wider widths and transitioning to narrower ones as the cleft gap reduced. Parents were trained in device placement, taping, and hygiene care, and follow-up was conducted both in clinic and remotely at regular intervals.

At 6 weeks of therapy, qualitative clinical changes in nasolabial anatomy were observed. The columella appeared longer and more centrally positioned along the midline. An apparent increase in nasal dome projection was observed, while the nostrils appeared more symmetric and increased in dimension, with an apparent reduction in septal deviation (Figure 2G–I). A clinically observable reduction in cleft gap width and closer lip approximation were also noted. On the cleft side, the nasal dome appeared elevated, and changes in the configuration of the alar cartilage were observed, with a more rounded nostril contour and greater apparent nasal symmetry. The bilateral nasal support provided by the NoseAlign device maintained contact with both nostrils, including the non-cleft side, where an apparent increase in columellar length and changes in nasal contour were also observed. Consistent use of the device was maintained throughout this treatment period. In this individual case, qualitative clinical assessment suggested a reduction in apparent cleft width, closer lip approximation, and more central apparent columellar positioning during treatment. The observed changes were associated with a clinically more balanced nasolabial appearance and closer approximation of the soft tissues before subsequent surgical repair. These observations remain qualitative and should not be interpreted as evidence that PLANA optimized surgical conditions or improved subsequent surgical outcomes.

No skin breakdown or adverse reactions were observed during the treatment period. Treatment use was consistently reported throughout the therapy period.

### 3.2. Case #2

**Figure 3:**
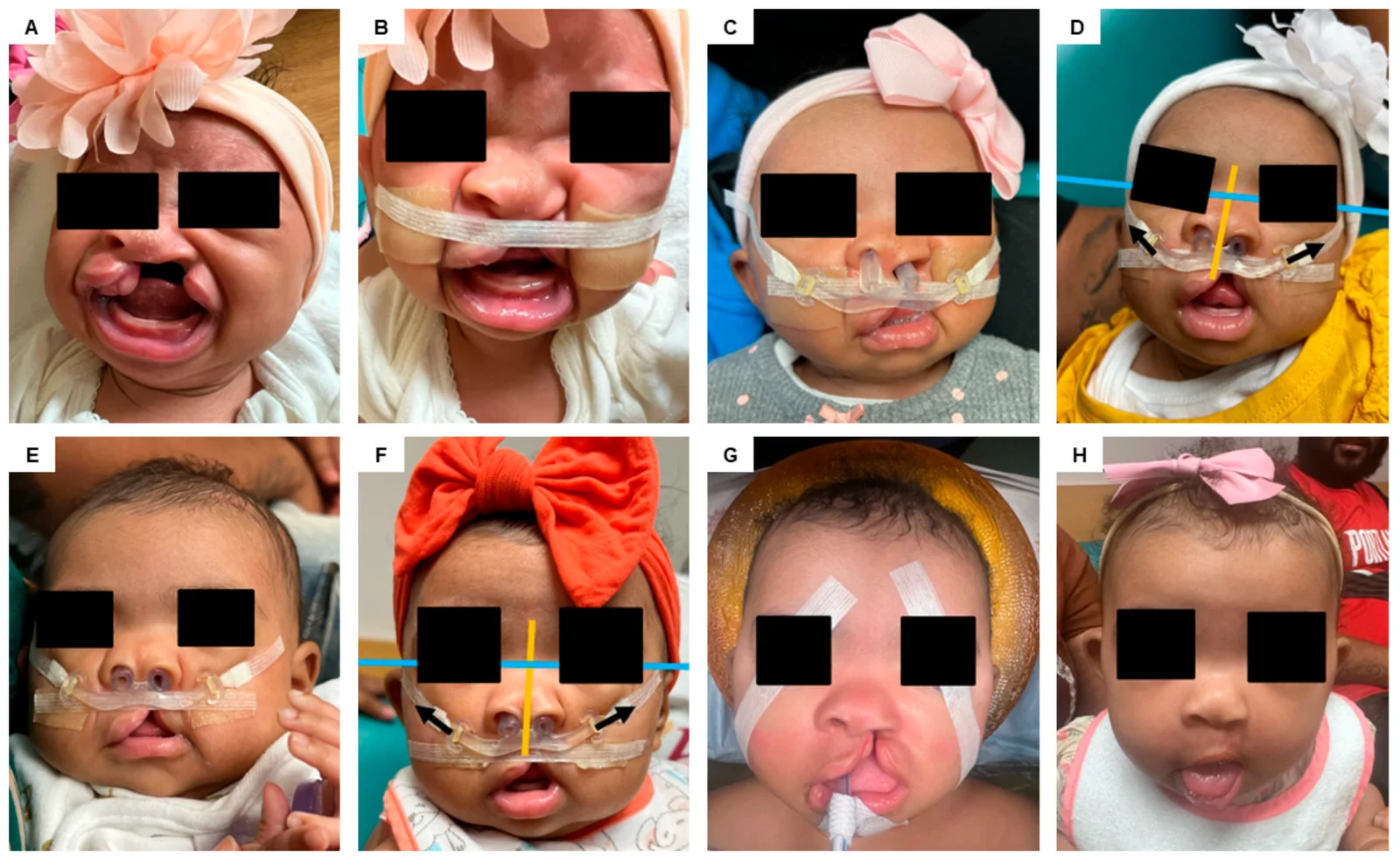
Treatment progression of an infant with unilateral complete cleft lip and palate using the PLANA protocol (**A**) Initial presentation. (**B**) LipAlign hydrocolloid adhesive taping initiated. (**C**–**F**) Application of the NoseAlign device, supported with Steri-Strips and 3/16-inch, 3.5 oz orthodontic elastics. Note the orientation of the interpupillary line (blue), the glabella–subnasal line (yellow), and the direction of elastic pulling forces (black arrows). (**G**) Later treatment-stage clinical appearance following PLANA treatment. (**H**) Facial appearance after PLANA treatment.

This case involves a female newborn diagnosed with complete left-sided unilateral cleft lip and palate (Figure 3A). At baseline, the infant presented with a wide cleft involving the lip, alveolus, and palate. Clinical features included distorted nasal cartilage, a deviated nasal septum to the right, shortened columella, flattened nasal dome, absence of a defined nostril on the cleft side, wide alar base width, and lateral displacement of both the alar base and nasal dome. The premaxilla was rotated toward the non-cleft side, contributing to marked nasal asymmetry.

Presurgical orthopedic treatment using the PLANA protocol was initiated during the neonatal period. Immediate LipAlign taping was applied (Figure 3B), followed by the progressive use of NoseAlign and LipAlign devices. Device dimensions were adjusted over time to accommodate facial growth and changes in cleft configuration (Figure 3C–F). This approach provided bilateral support to the nasal cartilages and may have contributed to the gradual uprighting and elongation of the columella.

By mid-treatment (Figure 3E,F), nasal dome molding was evident. The columella showed initial elongation, and the septum appeared more centrally aligned. The cleft-side nostril began to open, lift, and display a more rounded contour. The premaxilla appeared more favorably aligned, and the cleft gap was visibly reduced. Lip segments appeared more closely approximated, coinciding with the use of taping, with the tape positioned to approximate the orbicularis oris muscle segments and facilitate lip alignment. In the later treatment stage (Figure 3G,H), qualitative clinical changes were observed. The columella appeared longer and more centrally positioned, the nasal septum appeared more centrally aligned on clinical examination, and both nostrils appeared more symmetrically enlarged. Nostril shape and height appeared more symmetric. Nasal tip projection appeared increased, and an apparent reduction in the alveolar cleft gap was observed. The lip segments appeared closely approximated, with closer soft tissue approximation before surgical repair. Overall, qualitative clinical assessment suggested changes in nasal symmetry, apparent columellar length, and soft tissue alignment during the treatment period.

Following PLANA therapy, qualitative clinical assessment suggested a reduction in the apparent cleft width, closer lip approximation, narrowing and medialization of the alar base, and changes in the contour of the cleft-side alar cartilage. The nasal septum also appeared more centrally aligned on clinical examination. Additionally, the non-cleft side also showed qualitative clinical changes, including apparent columellar elongation and greater apparent nasal symmetry.

No adverse skin reactions or other adverse events were reported, and treatment adherence was reported as good throughout the treatment period.

### 3.3. Case #3

**Figure 4:**
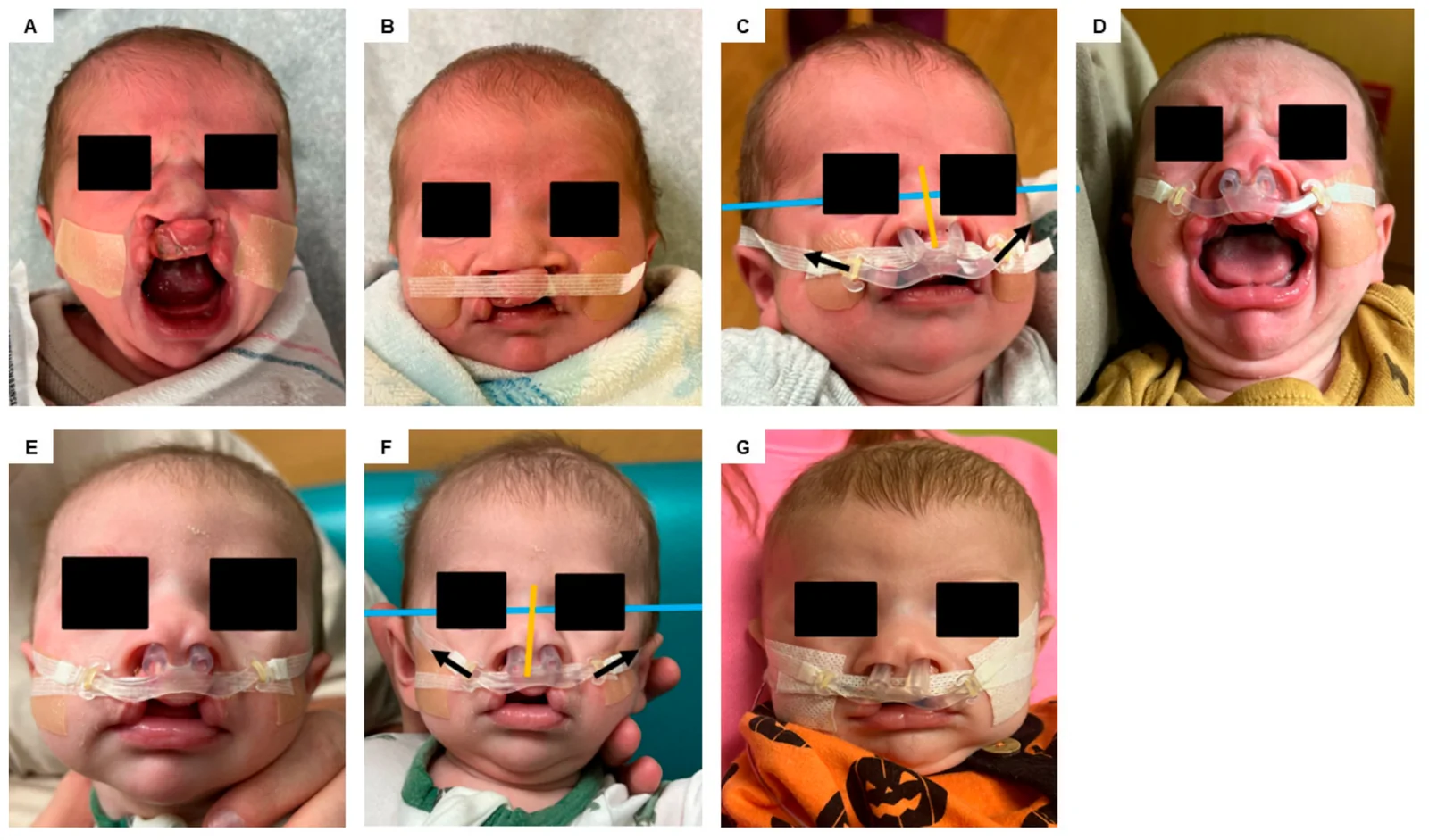
Presurgical orthopedic management of an infant with bilateral complete cleft lip and palate using the PLANA system (**A**) Initial presentation. (**B**) LipAlign taping initiated to apply medial traction to the lip segments. (**C**–**G**) Combined use of LipAlign and NoseAlign devices. The NoseAlign device is stabilized with Steri-Strips and 3/16-inch diameter, 3.5 oz orthodontic elastics. Note the orientation of the interpupillary line (blue), the glabella–subnasal line (yellow), and the direction of elastic pulling forces (black arrows).

An infant with bilateral complete cleft lip and palate was referred to the Alabama Cleft Center within the first weeks of life. At the initial presentation (Figure 4A), clinical findings included a wide bilateral cleft, severely displaced and protruded premaxilla, short columella, distorted nasal cartilage, absence of prolabium, increased alar base width, and marked nasal flattening. The alar bases were widened, and the nasal tip was poorly defined.

Treatment began using the PLANA protocol, starting with LipAlign hydrocolloid adhesive tapes (Figure 4B) and the NoseAlign device (Figure 4C). As therapy progressed, both devices were gradually replaced with the next sizes to match facial growth and changes in cleft configuration. The NoseAlign provided bilateral support to the nasal cartilages and was positioned to support columellar alignment (Figure 4C).

During treatment, LipAlign taping was applied with medial traction to the lip segments, with the aim of approximating the soft tissue margins and supporting prolabial positioning. Changes in the position of the premaxilla were also observed during treatment. Simultaneously, early bilateral shaping of the nasal domes was observed, with apparent changes in columellar length and nasal tip projection (Figure 4C).

In the final stages, there were qualitative clinical changes in soft tissue alignment: formation of a distinct columella, bilateral alar domes that appeared elevated and more symmetric, centralized prolabium with an apparent reduction in soft tissue tension, and a more balanced appearance of nostril shape, length, and width (Figure 4F,G). Compared with the baseline clinical appearance, qualitative assessment suggested changes in nasolabial morphology, including apparent changes in columellar definition, nasal symmetry and tip projection, narrowing of the alar base, reduction in the apparent cleft width, closer lip and prolabial approximation, and a more centrally aligned nasal septum. The bilateral nostrils also appeared more clearly defined and more similar in clinical appearance at the later treatment stage (Figure 4E–G).

No adverse skin reactions or other adverse events were reported, and treatment adherence was reported as good throughout the treatment.

### 3.4. Case #4

**Figure 5:**
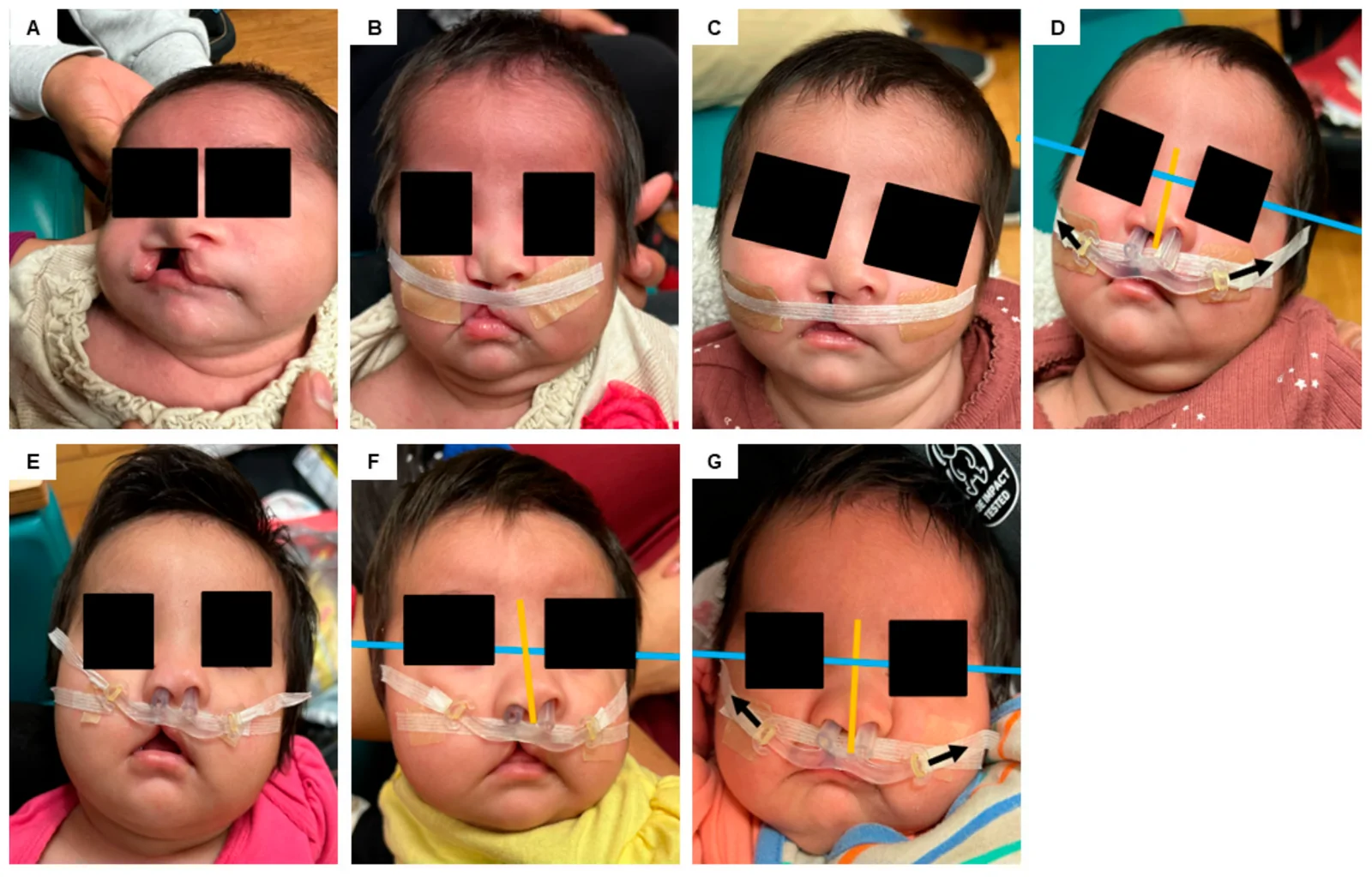
Presurgical orthopedic management of an infant with a complete right-sided unilateral cleft lip and palate using the PLANA system. (**A**) Initial presentation. (**B**,**C**) Application of LipAlign hydrocolloid adhesive taping. (**D**–**G**) Combined use of LipAlign and NoseAlign devices. Note the orientation of the interpupillary line (blue), the glabella–subnasal line (yellow), and the direction of elastic pulling forces (black arrows).

An infant with complete right-sided unilateral cleft lip and palate was referred to the Alabama Cleft Center during the neonatal period. At initial presentation (Figure 5A), clinical features included a wide cleft involving the right lip, alveolus, and palate; distorted nasal cartilage; and a nasal septum deviated to the non-cleft side, with a wide and flat nostril on the cleft side. There was marked nasal asymmetry, with a shortened columella deviated toward the non-cleft side, a flattened and deviated nasal tip (also toward the non-cleft side), and clinically observable displacement of the alar base. The nostril on the non-cleft side appeared narrowed, and the premaxillary segment was rotated toward the non-cleft side.

Treatment was initiated with LipAlign hydrocolloid adhesive taping (Figure 5B) to provide medial approximation of the lip segments during the early treatment phase. This was followed by application of the NoseAlign device (Figure 5D–G) according to the PLANA protocol, with progressive adjustments in both nasal and lip components tailored to accommodate facial growth and the changes in cleft configuration.

Medial traction from the Lip Align system was applied to the prolabial and lip segments, with the aim of supporting soft tissue approximation (Figure 5C). Nasal stenting with the Nose Align was associated with an apparent change in the configuration of the right alar dome (Figure 5D,E). Nasal tip projection and columellar length appeared more prominent by the mid-treatment phase (Figure 5D), with symmetrical vertical lifting forces applied by the nasal align device.

In the later stages of treatment (Figure 5F,G), qualitative clinical changes in nasal morphology were observed. The nasal tip projection appeared increased, a well-defined columella formed, the right (cleft-side) alar base elevated and appeared more medially positioned, and nostril widths appeared more balanced.

Compared with the initial clinical presentation, qualitative assessment suggested a reduction in the apparent cleft width, apparent columellar elongation, and greater apparent symmetry of the nasal domes. The nasal septum appeared more centrally aligned on clinical examination, and the prolabium appeared more clearly defined during the later stages of treatment. Additionally, there was closer lip approximation, narrowing and changes in the configuration of the alar base on the cleft side, and visible changes in the configuration of the alar cartilage on both sides. An apparent increase in columellar length on the non-cleft side was observed during treatment.

No adverse skin reactions or other adverse events were reported, and treatment adherence was reported as good throughout the treatment period.

## 4. Discussion

In this four-patient case series, treatment with the PLANA protocol was applied clinically, and no device-related complications or clinically significant skin intolerance were observed during the reported treatment period. Across the four cases, qualitative clinical observations included changes in nasal symmetry, columellar position and appearance, nasal contour, lip approximation, apparent cleft-width reduction, and overall nasolabial balance. These findings represent clinical observations made during treatment and should not be interpreted as objectively measured evidence of anatomical remodeling. In particular, changes in nostril morphology were observed in both the cleft and non-cleft sides in the reported cases. Although these observations may indicate favorable early changes in nasolabial configuration, the present study was not designed to quantify the magnitude of these changes or determine their clinical significance. Whether such changes improve subsequent surgical outcomes was not evaluated.

The qualitative clinical observations described in this series are consistent with the general objectives of PSIO, which aim to optimize early anatomical relationships before surgical repair [3,4,5]. Presurgical interventions are intended to approximate the tissues around the cleft and improve the anatomical conditions before surgical cleft repair rather than provide definitive correction of the cleft deformity [3,4,5]. Within this context, the present case series provides preliminary clinical experience regarding the application and feasibility of the PLANA protocol in infants with unilateral or bilateral cleft lip and palate. These observations should be regarded as descriptive and hypothesis-generating rather than as evidence of treatment efficacy.

The qualitative clinical observations in this case series are similar to anatomical changes previously described in report of the PLANA technique. For example, Multani et al. [11] reported quantitative improvements in nasal symmetry following PLANA therapy. The observations in the present series appear directionally similar to those findings; however, direct comparison is limited because the current study did not use standardized quantitative anthropometric measurements or a validated scoring system. Accordingly, the present observations should be considered complementary clinical experience rather than independent confirmation of the quantitative effects reported previously.

The bilateral improvement in nostril morphology observed in this series may be related to the design characteristics of the PLANA protocol, particularly the incorporation of the bilateral NoseAlign component. By applying controlled molding forces simultaneously to both sides of the nasolabial complex, the device may facilitate more balanced adaptation of the nasal structures and contribute to progressive improvement in nasal contour, columellar position, and nostril symmetry. This bilateral approach may be particularly relevant in cleft conditions, where asymmetrical tissue forces contribute to distortion of the nasal framework. Nevertheless, this proposed mechanism remains hypothetical, as biomechanical parameters, including force distribution and tissue-level responses, were not directly evaluated in the present study.

The present study was not designed to compare PLANA with NAM, as all infants received PLANA treatment and no contemporaneous control group was included. Accordingly, references to NAM are intended solely to contextualize the current findings within the existing literature, particularly the comparative investigation reported by Multani et al. [11]. No conclusions regarding comparative effectiveness, superiority, or clinical advantage of PLANA over NAM can be established from the present data. Future prospective comparative investigations using standardized outcome measures will be required to determine the relative benefits of different presurgical orthopedic approaches.

Although asymmetric clefts may present challenges for proper placement of the NoseAlign device, early initiation of treatment may be advantageous and should be considered when clinically appropriate. Early intervention may facilitate device adaptation and progressive molding. Furthermore, asymmetric elastic traction plays an important role in the gradual correction of cleft-related deformities.

An additional clinical consideration is the potential importance of treatment timing during the neonatal period. In this series, infants who initiated PLANA treatment within the first 1.5 weeks appeared to demonstrate favorable treatment progression; however, this observation was based on clinical experience within a limited case series and should be considered hypothesis-generating rather than evidence of an optimal treatment window. The potential rationale for early intervention relates to the increased adaptability of neonatal cartilage during the early postnatal period, which has been associated with transient biological characteristics that may enhance responsiveness to external molding forces [9,10]. Moreover, early nasal cartilage molding is particularly important because neonatal cartilage has been reported to contain higher levels of hyaluronic acid, which may contribute to increased tissue plasticity during this period [9,10]. Delays in initiating NAM therapy may reduce the opportunity to fully benefit from this critical period. Further investigations are required to determine whether the timing of PLANA initiation influences treatment outcomes.

From a clinical perspective, the extraoral design of PLANA may offer practical advantages by eliminating the need for intraoral components traditionally used in appliance-based presurgical orthopedic approaches. The absence of intraoral components may simplify treatment management, improve infant tolerance, and reduce some practical challenges associated with intraoral appliances. These features may have practical implications for treatment administration and caregiver burden, although these outcomes were not formally assessed in the present series, which are important considerations in presurgical orthopedic therapy. However, the potential impact of appliance design on compliance, treatment burden, and patient-reported outcomes requires objective evaluation in future comparative studies.

Overall, this case series describes the clinical application of the PLANA protocol and provides preliminary qualitative observations of changes in nasolabial morphology during treatment. The findings are encouraging and broadly comparable in direction to previously reported observation; however, the descriptive design, small sample size, absence of a control group, lack of standardized quantitative measurements, and absence of formal reliability assessment substantially limit the strength of inference. The present findings should therefore be interpreted as preliminary clinical experience rather than evidence of treatment efficacy or comparative benefit. These observations provide a rationale for prospective studies designed to determine the reproducibility, magnitude, and clinical relevance of PLANA-associated anatomical changes.

### 4.1. Clinical Considerations During PLANA Treatment Planning: Natal Tooth Observation

An additional clinical observation encountered during PLANA treatment planning involved a natal tooth in a newborn with cleft lip and palate. This infant was not included in the four-patient PLANA case series and is presented separately to illustrate a potential clinical consideration during early cleft management.

**Figure 6:**
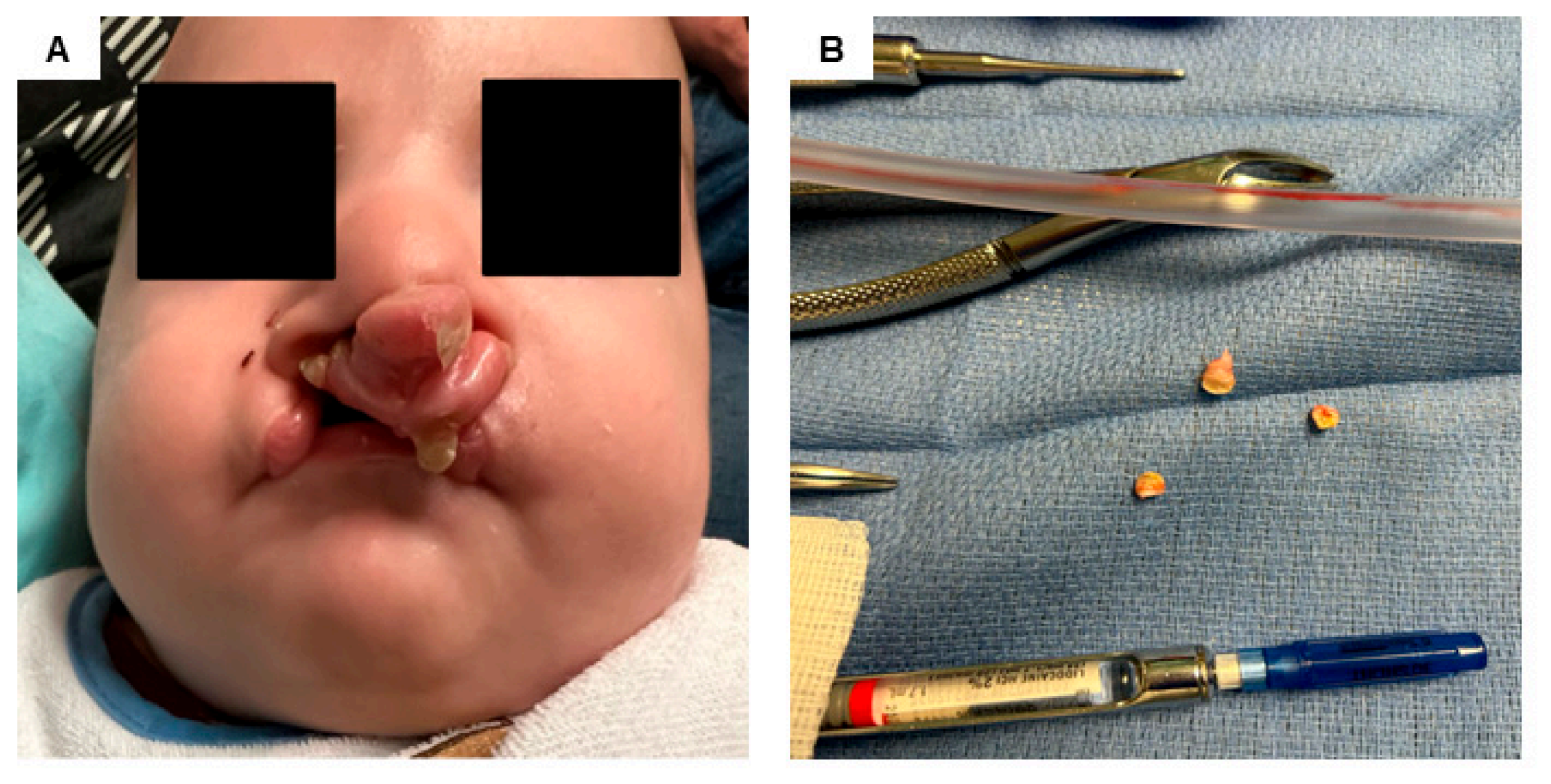
Clinical management consideration of a natal tooth in a newborn with cleft during presurgical cleft care. (**A**) Clinical photograph showing the presence of a natal tooth located within the cleft region in a newborn. (**B**) Clinical appearance following extraction of the natal tooth after administration of vitamin K and local anesthesia with lidocaine, followed by curettage of the extraction socket.

Natal teeth may be encountered in infants with cleft lip and palate and, depending on their location, mobility, and relationship to the cleft, may influence presurgical treatment planning [20,21]. Although PLANA does not require an intraoral appliance, early examination of the oral cavity remains important to identify dental findings that may require individualized management. In the present observation, the natal tooth was extracted following administration of vitamin K and local anesthesia with lidocaine, with subsequent curettage of the socket (Figure 6A,B). This observation is presented as a clinical consideration rather than as an outcome of PLANA treatment.

### 4.2. Limitations and Future Direction

The present study has important methodological limitations that substantially restrict the strength and generalizability of its conclusions. First, this was a descriptive case series involving only four infants. The small sample size prevents meaningful assessment of variation in treatment response according to cleft type, severity, or other patient-level characteristics and substantially limits generalizability. The study therefore provides preliminary clinical experience and hypothesis-generating observations rather than evidence of treatment efficacy, comparative effectiveness, or generalizable clinical recommendations.

Another important limitation is the absence of a contemporaneous NAM-treated or another control group. Consequently, this study cannot determine whether the observed clinical changes differ from those achievable with NAM or other presurgical orthopedic approaches. Any discussion comparing PLANA with NAM is based exclusively on previously published studies and should therefore be interpreted as indirect comparisons rather than evidence of comparative effectiveness. Because standardized quantitative outcome measurements were not collected and the study comprised only four descriptive cases, formal statistical comparison was not performed. Consequently, the magnitude and statistical significance of pre- to post-treatment changes cannot be determined. The case-series design may introduce selection bias. The patients included in this report may not represent the full spectrum of cleft severity, and factors such as cleft morphology, individual growth patterns, treatment compliance, and clinician experience may have influenced the observed outcomes. In addition, palatal morphology was not systematically evaluated using standardized digital methods, and palatal and alveolar changes were not quantified using standardized objective measures. Furthermore, because treatment evaluation was performed within the same clinical setting, the potential influence of operator-related factors cannot be excluded. Because the qualitative clinical assessments were performed without formal interobserver or intraobserver reliability testing, potential observer-related variability cannot be excluded. The possibility that some of the reported changes reflect subjective interpretation rather than objectively measurable anatomical change therefore remains. Future studies should use predefined assessment criteria, blinded or independent assessors where feasible, and formal interobserver and intraobserver reliability testing to establish the reproducibility of clinical assessments. Future studies should incorporate validated assessment protocols with predefined criteria and reliability analysis to improve reproducibility and methodological rigor.

Another limitation is the absence of standardized quantitative outcome assessments. Although clinical improvements in cleft width reduction, premaxillary alignment, and nasolabial morphology were observed, objective measurements were not systematically performed. The absence of three-dimensional digital analysis, serial intraoral scanning, and validated quantitative assessment methods limits the ability to precisely evaluate the magnitude of anatomical changes and objectively compare outcomes across patients or with other treatment modalities. Future studies incorporating three-dimensional imaging, digital intraoral scanning, photogrammetric analysis, and standardized measurement protocols may provide a more rigorous assessment of alveolar, palatal, and nasolabial changes during PLANA therapy and a more comprehensive evaluation of treatment outcomes.

Finally, the limited follow-up period prevents evaluation of the long-term stability of the achieved anatomical changes and their potential influence on subsequent surgical outcomes and facial growth. Since presurgical orthopedic interventions are intended to modify early anatomical relationships before primary surgery, long-term studies are required to determine whether the initial improvements are maintained and whether they translate into clinically meaningful benefits. Larger prospective studies with extended follow-up, appropriate control groups, objective three-dimensional outcome measurements, and standardized quantitative assessment methods are necessary before definitive conclusions regarding the effectiveness and clinical advantages of PLANA can be established.

Despite these limitations, this case series provides preliminary clinical experience with the PLANA technique and contributes to the limited available evidence regarding its feasibility and early outcomes. The preliminary qualitative observations from this study indicate favorable early clinical changes, although these findings should be interpreted cautiously because of the limited evidentiary value of the case-series design. Importantly, the procedure was complication-free in our series, with no reported adverse effects or allergic reactions. These findings provide a foundation for future prospective multicenter studies aimed at validating the reproducibility of PLANA outcomes across different clinical settings and evaluating its potential clinical benefits using objective outcome measurements and long-term follow-up.

## 5. Conclusions

This four-case series describes the clinical application of the PLANA technique and provides preliminary qualitative observations regarding nasolabial morphology and clinical treatment feasibility. Given the descriptive nature and limited sample size of this case series, these findings should be interpreted as preliminary and hypothesis-generating rather than confirmatory, and conclusions regarding treatment effectiveness cannot be established. Larger prospective studies incorporating objective anthropometric measurements, quantitative image analysis, appropriate comparison groups, statistical analyses, and long-term follow-up are required to determine the clinical benefits, reproducibility, and long-term outcomes of the PLANA approach.

## Figures and Tables

**Table 1:** Baseline Demographic and Clinical Characteristics of the Four Infants.

Patient	Age (Days)	Cleft Type	Columella Length (Qualitative)	Baseline Nasal Symmetry (Qualitative)	Baseline Nasal Septal Orientation (Qualitative)	Additional Baseline Anatomical Findings (Qualitative)	Treatment	Wear Time (h/Day)	Treatment Duration (Weeks)
1	7	Unilateral left CLP	Reduced	Asymmetric	Deviated toward the right	Wide alar base, narrow nostril	PLANA	20–22	22
2	8	Unilateral left CLP	Reduced	Asymmetric	Deviated toward the right	Wide alar base, absent nostril on cleft side, rotated premaxilla	PLANA	20–22	21
3	7	Bilateral CLP	Reduced	Asymmetric	Deviated	Displaced, protruding premaxilla, prolabium deficiency	PLANA	20–22	22
4	9	Unilateral right CLP	Reduced	Asymmetric	Deviated toward the left	Wide alar base, premaxilla rotated to the left	PLANA	20–22	23

Data represent qualitative clinical observations recorded at baseline; standardized anthropometric measurements were not performed. Abbreviation: CLP, cleft lip and palate; h, hour.

**Table 2:** Qualitative Clinical Observations Following PLANA Therapy.

Patient	Qualitatively Observed Columellar Appearance	Qualitatively Observed Nasal Symmetry	Qualitatively Observed Nostril Morphology	Qualitatively Observed Nasal Septal Orientation	Qualitatively Observed Lip Approximation	Qualitatively Observed Cleft Width	Adverse Events
1	Appeared longer	Greater apparent symmetry on clinical assessment	Appeared more symmetric	Appeared more centrally aligned	Closer approximation	Apparent reduction	None
2	Appeared longer	Greater apparent symmetry on clinical assessment	Appeared more symmetric	Appeared more centrally aligned	Closer approximation	Apparent reduction	None
3	Appeared longer	Greater apparent symmetry on clinical assessment	Appeared more symmetric	Appeared more centrally aligned	Closer approximation	Apparent reduction	None
4	Appeared longer	Greater apparent symmetry on clinical assessment	Appeared more symmetric	Appeared more centrally aligned	Closer approximation	Apparent reduction	None

All outcomes represent qualitative clinical observations based on serial clinical photographs and routine clinical examinations. No validated anthropometric measurements, quantitative image analysis, three-dimensional measurements, or standardized clinical scoring system were used. Accordingly, the reported findings do not represent numerical estimates of anatomical change.

## Data Availability

The data supporting the findings of this study are presented within the manuscript. Additional patient-level data are not publicly available because of privacy and ethical restrictions related to the protection of patient confidentiality.
